# 388. Co-Testing: Increasing HIV Testing in Patients Undergoing Sexually Transmitted Infection Evaluation in a Large Integrated Healthcare Network

**DOI:** 10.1093/ofid/ofae631.123

**Published:** 2025-01-29

**Authors:** Allan M Seibert, Michelle M Matheu, Whitney Buckel, Tamara Moores Todd, Caroline MD Vines, Joseph Bledsoe, Adam Balls, James Hellewell, Randall J Smout, Bert K Lopansri, Valoree K Stanfield, Vanessa R Wormser, Matt Gwiazdon, Park Willis, Anthony Wallin, Adam Hersh, Payal K Patel, Brandon J Webb, Eddie Stenehjem

**Affiliations:** Intermountain Health, Murray, UT; Intermountain Healthcare, Salt Lake City, Utah; Intermountain Health, Murray, UT; Intermountain Health, Murray, UT; Intermountain Health, Murray, UT; Intermountain Health, Murray, UT; Intermountain Health, Murray, UT; Intermountain Health, Murray, UT; Intermountain Health, Murray, UT; Intermountain Healthcare, Salt Lake City, Utah; Intermountain Healthcare, Salt Lake City, Utah; Intermountain Health, Murray, UT; Beth Israel Deaconess Medical Center, Boston, Massachusetts; Intermountain Healthcare, Salt Lake City, Utah; Intermountain Healthcare, Salt Lake City, Utah; University of Utah School of Medicine, Salt Lake City, UT; Intermountain Healthcare, Salt Lake City, Utah; Intermountain Healthcare, Salt Lake City, Utah; Division of Infectious Diseases, University of Colorado, Aurora, Colorado

## Abstract

**Background:**

Utah ranks last among states in the percentage (26.5%) of adults ever tested for Human Immunodeficiency Virus (HIV). We hypothesized that linking “co-testing” for HIV to evaluations for sexually transmitted infections (STIs) in Urgent Care (UC) and Emergency Departments (ED) would increase HIV screening. We implemented a multifaceted intervention in the UCs and EDs of our integrated health system and evaluated its impact on HIV co-testing.

**Figure:**
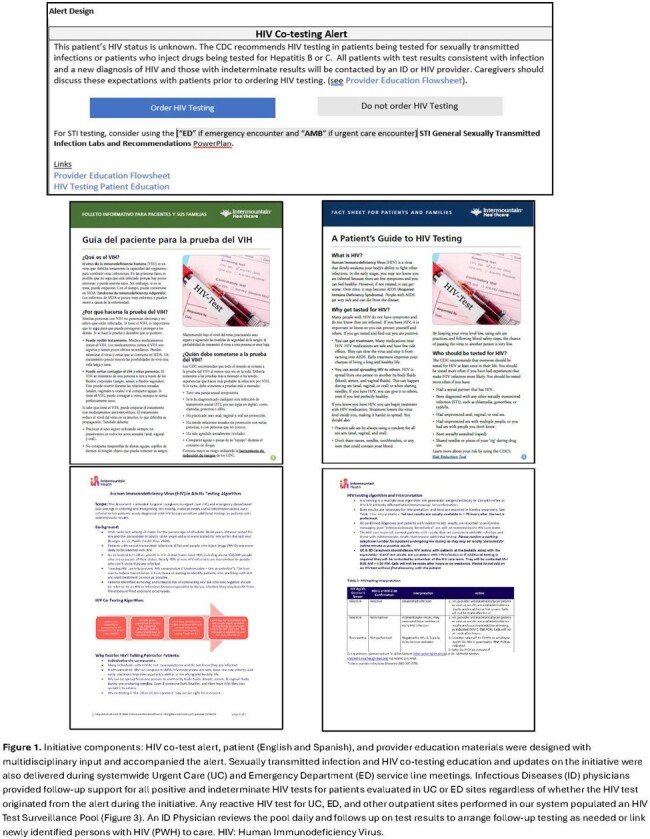

**Methods:**

The initiative included education and HIV test follow-up support along with an electronic medical record-embedded alert **(Figure 1)**. The alert surfaced if gonorrhea (GC), chlamydia (CT), or other STI testing was ordered for patients ≥ 18 years with unknown HIV status and encouraged HIV testing if not already ordered. We sequentially implemented the initiative in 26 UC and 22 ED sites from April 2023 – December 2023. We used interrupted time series analysis to evaluate HIV co-testing rates among eligible GC/CT testing encounters in pre-implementation (April 1^st^, 2022 – March 31^st^, 2023) and post-implementation (April 1^st^, 2023 – March 31^st^, 2024) UC and ED cohorts. We compared the observed rate to the predicted counterfactual (had no intervention taken place). We also assessed co-testing by social determinants of health (SDOH) categories, alert volume, and system impact on HIV testing and diagnoses.

**Figure:**
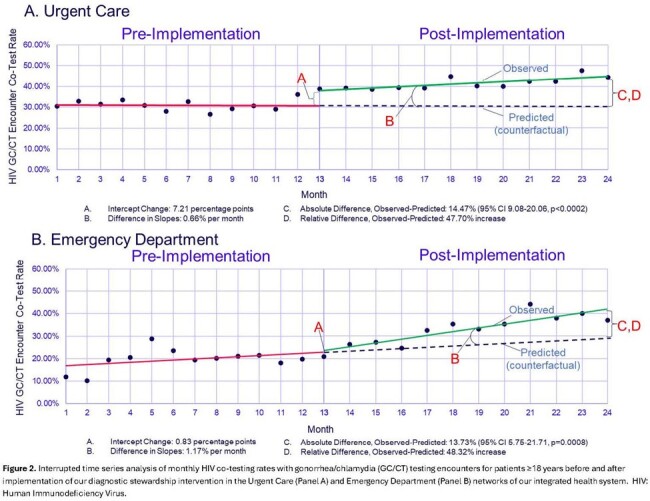

**Results:**

During the 24-month study, there were 19,232 GC/CT test encounters: 5,730 UC/2,524 ED pre-implementation and 7,696 UC/3,282 ED post-implementation. In UC, the increase in the observed HIV GC/CT co-test rate vs the predicted was 14.5 percentage points (95% CI 9.08-20.06, p< 0.001) **(Figure 2)**. In the ED, the increase in the observed HIV GC/CT co-test rate vs the predicted was 13.7 percentage points (95% CI 5.75-21.71, p< 0.001). HIV GC/CT co-testing varied by SDOH **(Table 1)**. 4,704 alerts fired (54% UC/46% ED) and resulted in 730 HIV co-tests (225 UC/505 ED), representing a 15.5% engagement rate. Of these 730 alert-initiated HIV co-tests, 5 (0.7%) revealed new HIV diagnoses, representing 29% of all HIV diagnoses in UC and ED during the post-implementation period **(Figure 3).**

**Figure:**
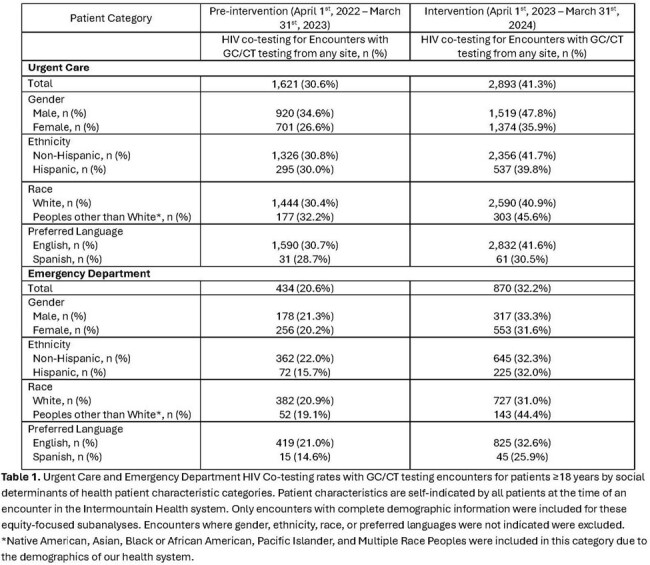

**Conclusion:**

HIV co-testing in our UC and ED sites increased during a multifaceted initiative. New HIV diagnoses occurred in UC and ED as a result of HIV testing ordered via our alert.

**Figure:**
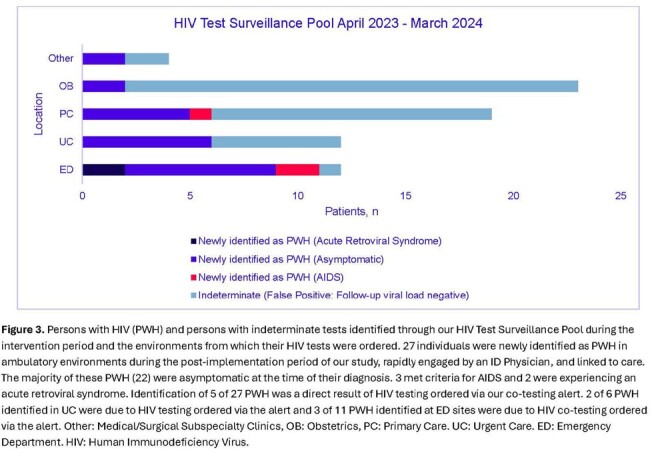

**Disclosures:**

**Bert K. Lopansri, MD, D(ABMM), FIDSA**, Cepheid: Grant/Research Support|Seegene: Advisor/Consultant

